# Primary Sjögren's Syndrome Presenting as Cerebral Venous Thrombosis: A Rare Case

**DOI:** 10.7759/cureus.28772

**Published:** 2022-09-04

**Authors:** Mohan Sonu Chandra, Monisha G A, Ravi Kiran M

**Affiliations:** 1 Internal Medicine, Mysore Medical College and Research Institute, Mysuru, IND

**Keywords:** keratoconjunctivitis sicca, multiple abortions, headache, cerebral venous thrombosis, sjogrens syndrome

## Abstract

Sjogren’s syndrome is a late-onset, slowly progressing autoimmune disease characterized by the destruction of the exocrine glands by lymphocytic infiltration, resulting in dry mouth (xerostomia) and dry eyes (keratoconjunctivitis sicca). Sjögren's syndrome may be associated with various autoimmune diseases, including systemic lupus erythematosus, rheumatoid arthritis, and systemic sclerosis. We report a case of a 34-year-old female who delivered a live baby 20 days ago. She presented in a postictal state after two episodes of tonic-clonic movements of limbs with altered sensorium with a history of headache for seven days. Further evaluation revealed that the subject had a history of multiple abortions and grittiness in her eyes. MRI showed signs of infarction in the left parietal lobe and magnetic resonance venography (MRV) suggested cavernous venous thrombosis. After an unwavering effort to rule out alternate causes, the rare correlation between primary Sjogren’s syndrome and cerebral venous thrombosis was considered. Additional investigations were performed, which showed the patient to be positive for Anti SS-A (Ro52), Anti SS-B (La), and anti-centromere antibodies. The patient gradually improved with anti-edema measures and steroids and was discharged by day nine. We present this case to emphasize the neurological manifestation of Sjogren’s syndrome, which may present as cerebral venous thrombosis.

## Introduction

Sjogren's syndrome is a chronic autoimmune condition characterized by the lymphocytic infiltration of the exocrine glands and increased B cell hyperactivity. The underlying pathogenesis may include hypergammaglobulinemia and serum autoantibodies, including antinuclear antibodies, rheumatoid factor, cryoprecipitate immunoglobulins, and antibodies against two ribonucleoprotein complexes named Ro/Sjögren's-syndrome-related antigen A (SS-A) and La/Sjögren's-syndrome-related antigen A (SS-B) are considered the hallmark of the disease [1,2]. The clinical presentation varies from dryness of mucosal surfaces to extraglandular manifestations as systemic diseases. The dryness of mucosal surfaces is predominantly a result of the immune activity in the exocrine glands [3]. The prognosis is primarily determined by the systemic involvement of the disease [4]. Sjogren's syndrome occurs mainly in middle-aged women, and affected women are more likely to have complicated pregnancies [5]. Distal renal tubular acidosis, which results in electrolyte imbalance and hypokalemic paralysis, might be caused by renal impairment in Sjogren's syndrome [6]. Neurological implications of Sjögren's are rare but commonly involve the peripheral nervous system [7]. Atypical neurological complications may prevent early diagnosis [8]. The common CNS manifestations include myelitis and small vessel vasculitis [9]. To date, only a few cases of cerebral venous thrombosis (CVT) with the presentation of Sjogren's syndrome have been reported. It was associated with myeloradiculopathy [10].

## Case presentation

A 34-year-old female who delivered a live baby 20 days ago presented to the medical emergency in a postictal state with two episodes of general tonic-clonic movements of the limbs with loss of consciousness, following which she regained consciousness. She had a history of headache for the past week, which was throbbing in nature, present throughout the day, and more severe in the frontal region. It was not associated with nausea, vomiting, photophobia, tinnitus, or dizziness. Her attendees gave the history.

**Figure 1:**
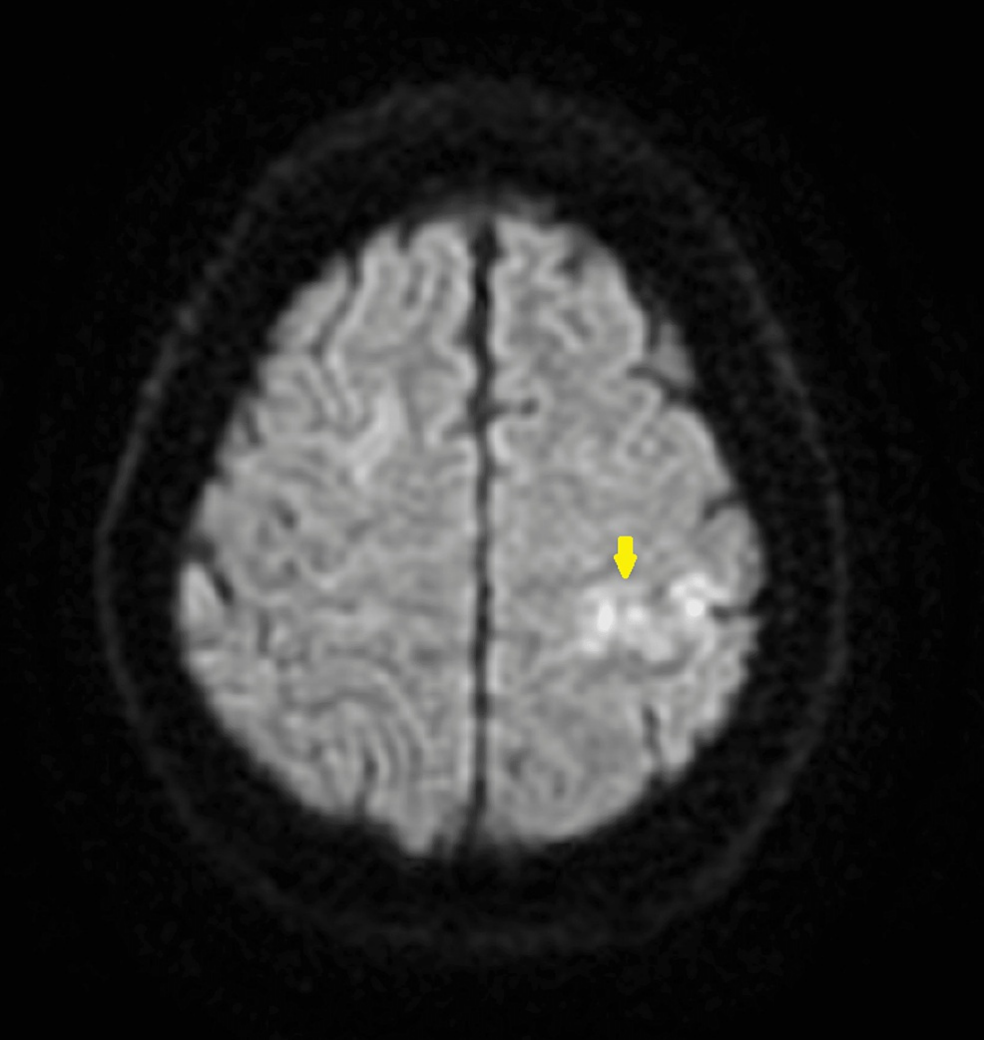
Axial view of MRI DWI sequence showing diffusion restriction signifying infarction MRI, magnetic resonance imaging; DWI, diffusion weighted imaging

**Figure 2:**
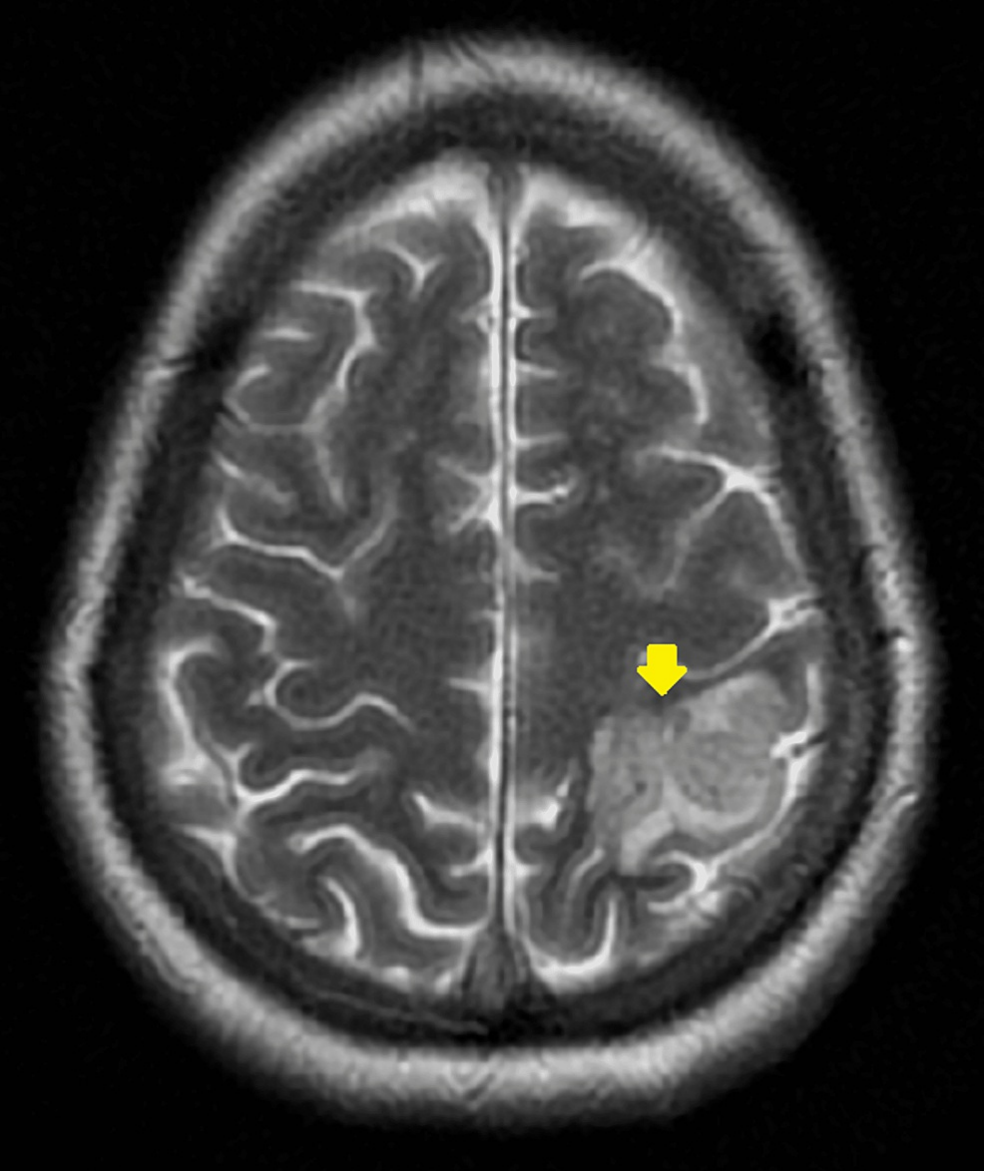
Axial view of T2 weighted MRI depicting hyperintensity in left parietal lobe MRI, magnetic resonance imaging

**Figure 3:**
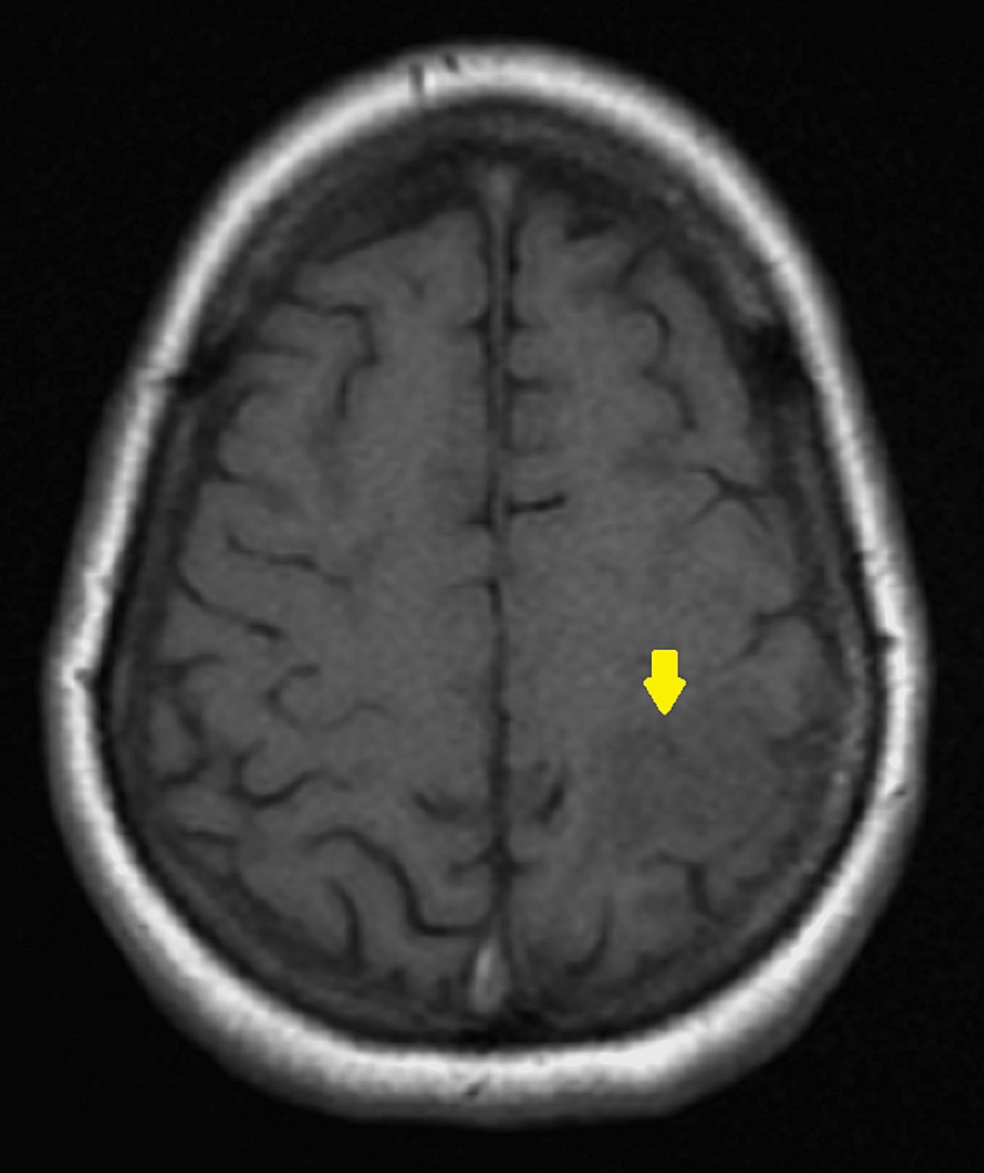
Axial view of T1 weighted MRI depicting hypointensity in left parietal lobe MRI, magnetic resonance imaging

**Figure 4:**
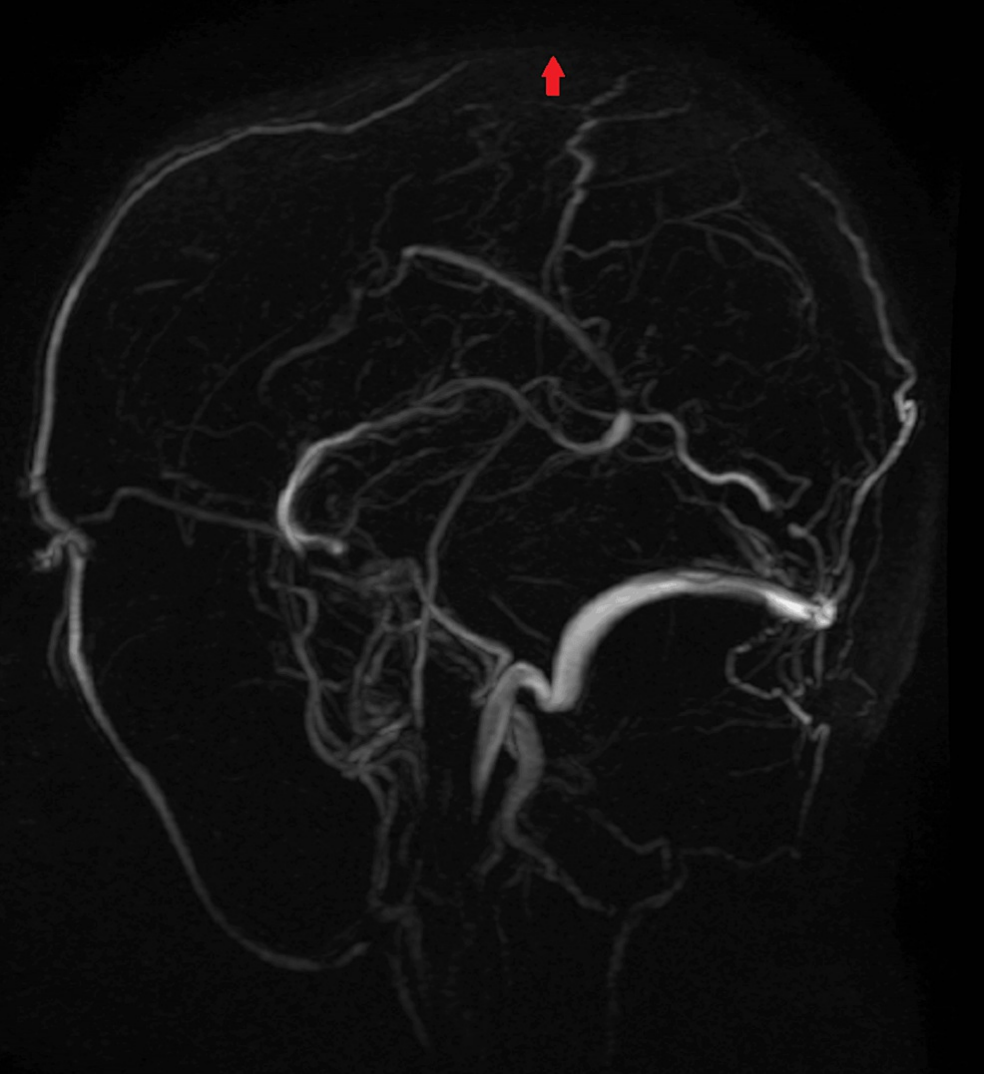
Magnetic resonance venogram depicting superior sagittal sinus thrombosis

**Figure 5:**
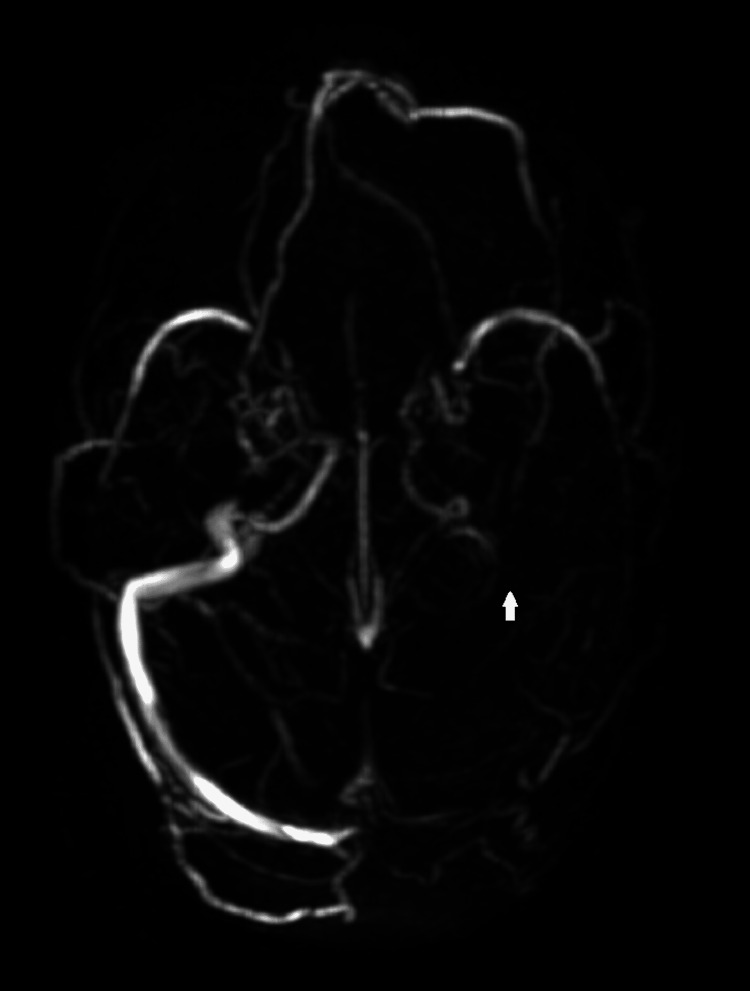
Magnetic resonance venogram depicting thrombosis of left sigmoid and transverse sinuses

Bilateral grade 1 pitting edema and frothing from the mouth was noticed on physical examination. Upon evaluation of the CNS, a Glasgow Coma Scale (GCS) score of 5 was recorded (E2V2M1). Pupils appeared bilaterally equal and were responsive to light. There was a bilateral plantar extensor response. The respiratory system was normal with bilateral normal vesicular breath sounds upon auscultation. The cardiovascular and abdominal examination showed no abnormality. The patient was initially managed by giving a loading dose of antiepileptic and was put on a maintenance dose. The consciousness level subsequently improved. Fundus examination revealed features of papilledema bilaterally. Magnetic resonance imaging (MRI) of the brain showed features of infarct in the left parietal lobe (Figures 1, 2, 3), and magnetic resonance venogram (MRV) showed thrombosis of the superior sagittal sinus (Figure 4) and left sigmoid and transverse sinuses (Figure 5). Injection ceftriaxone 1 g twice daily, subcutaneous low molecular weight heparin 60 μg twice daily, and injection mannitol 100 ml three times a day were started and continued for five days.

On day two of hospitalization, the consciousness level of the patient improved with a paucity of movements on the right limbs and the persistence of mild headaches. There was a normal deep tendon reflex on the right side. Upon further evaluation, she had a history of three abortions, two at home and one at a hospital, all at three months of gestation. The patient has had two live births; the first baby is three years old. She complained of experiencing grittiness in the eyes for two years with no history of ptosis, ophthalmoplegia, and diplopia. The patient is a non-alcoholic and a non-smoker with a BMI of 20.1. She has no history of fever, breathlessness, urinary symptoms, aural symptoms, dryness of mouth, difficulty in swallowing, previous significant headache events, seizures, and history of any toxin ingestion.

Routine investigations and further evaluation showed the patient to be anemic with an antinuclear antibody (ANA) profile positive for SS-A, SS-B, and anti-centromere antibodies as depicted in Tables 1, 2. Total counts, renal function tests, liver function tests, thyroid function tests, and urine routines were within normal limits.

**Table 1:** Laboratory values Hb, hemoglobin; HCT, hematocrit; MCV, mean corpuscular volume; MCH, mean corpuscular hemoglobin; MCHC, mean corpuscular hemoglobin concentration; ESR, erythrocyte sedimentation rate; INR, international normalized ratio; VDRL, venereal disease research laboratory test

Investigation	Normal	On admission
Hb (g/dL)	12.0-16.0	10
HCT	36.0%- 48.0%	30.4%
MCV (/fL)	80.0-99.0	67.5
MCH (/pg)	26.0-32.0	17.8
MCHC (/dL)	32.0-36.0	28.9
ESR (mm/hr)	0-29	54
INR	≤1.1	1.2
HIV, Hepatitis C, VDRL	-	Negative
Bleeding time (minutes)	2-7	4
Clotting time (minutes)	8-15	9
D-Dimer (ng/mL)	≤250	230
Protein C (activity; %)	70-140	112
Protein S (activity; %)	65 -130	96
Homocysteine (µmol/L)	4-12	7
Factor V Leiden	Negative	Negative

**Table 2:** Antibody profile C-ANCA, antineutrophil cytoplasmic autoantibody, cytoplasmic; P-ANCA, perinuclear anti-neutrophil cytoplasmic antibodies; Anti SS-A, anti–Sjögren's-syndrome-related antigen A; Anti SS-B: anti–Sjögren's-syndrome-related antigen B

Antibody	Result
Anti SS-A (Ro52) antibody, IgG	3 U (positive)
Anti SSB (La) antibody, IgG	2 U (positive)
Anti-centromere antibody	1:40 (weakly positive)
Rheumatoid factor	Negative
Antimitochondrial antibody	Negative
C-ANCA	Negative
P-ANCA	Negative
IgG and IgM anticardiolipin antibodies (aCL)	Negative
IgG and IgM anti-β2 glycoprotein-1 antibodies	Negative
Lupus anticoagulant (LA)	Negative

Other coagulative disorders were ruled out. Labial salivary gland biopsy showed lymphocytic infiltration and bilateral Schirmer test was <5mm in five minutes. The patient was diagnosed with Sjogren's syndrome on day four of hospitalization. The patient's condition gradually improved with anti-edema measures and steroids, and there was an improvement in power in a week. The patient was discharged on day nine and could walk independently. Anticoagulants and steroids, along with antiepileptics, were prescribed and advised for follow-up.

## Discussion

CVT is characterized by a total or partial obstruction of the major cerebral venous sinuses or the smaller feeding cortical veins (cortical vein thrombosis). It is a leading cause of stroke in young people, with a mean age of 33 years with a two-third female prevalence. Clinicians frequently overlook or misdiagnose CVT because it can mirror other acute neurological diseases and is only detectable with accurate and timely brain imaging [11]. 

Sjogren's syndrome is an autoimmune condition associated with human leukocyte antigen-DR isotype (HLA DR 3), more prevalent in women with a female:male ratio of 9:1. It is characterized by infiltration of lymphocytic cells in exocrine glands anywhere in the body [7]. Extraglandular manifestations are common, and they may present as arthritis, Raynaud's phenomenon, renal tubular acidosis, vasculitis, and lymphoma. CNS may also be involved, which may present as myelitis [12], small-vessel vasculitis [13], and arterial strokes [9,14].

Sjogren's syndrome may be associated with the presence of antiphospholipid antibodies (aPL), which may be one of the causes of thromboembolic events and fetal loss [15]. Thromboembolic events can also occur in the absence of aPL. According to Hughes and Khamashta, there might be three different possibilities to explain thromboembolic events occurring in seronegative patients: (i) a seronegative phase of a previously positive aPL, (ii) conventional laboratory testing failing to detect cases with antibodies directed against different phospholipids or protein cofactors, (iii) the presence of a different coagulopathy [16].

Patients with symptoms similar to aPL syndrome (APS) but with serology tests negative for anti-beta 2 glycoprotein, anticardiolipin, and lupus anticoagulant antibodies are classified as seronegative APS. Some patients might test positive for other non-specific antibodies, which include phosphatidic acid, phosphatidylserine, and phosphatidylinositol [17], which are not routinely assessed in the laboratories. Hence, APS cannot be ruled out as a cause for CVT in our patients.

Pregnancy complications are quite common with Sjögren's syndrome. According to studies, women with Sjögren's syndrome frequently experience unfavorable pregnancy outcomes like spontaneous abortion and fetal loss, stillbirths, intrauterine growth restriction, premature deliveries, and congenital heart blocks. Prenatal counseling is required for women with this underlying autoimmune condition to discuss all the hazards and the need for disease management before conception [4].

In cases of headaches with seizures, rare causes like connective tissue disorders should also be considered [18]. Our patient presenting with CVT had a normal coagulation profile, hydration status, protein c, and protein s levels, history of grittiness in the eyes, and history of previous abortions, leading us to disregard postpartum CVT and evaluate for connective tissue disorders.

## Conclusions

Practitioners should be aware of the rare presentation of Sjogren's as CVT. In any CVT patient, clinicians must consider a differential diagnosis of Sjogren's even if the patient tests negative for the specific diagnostic markers. If possible, the screening test must be repeated to confirm the serology. Headaches with seizures may be associated with connective tissue disorders.

## References

[1] Mavragani CP, Moutsopoulos HM (2010). The geoepidemiology of Sjögren's syndrome. Autoimmun Rev.

[2] Routsias JG, Tzioufas AG (2010). Autoimmune response and target autoantigens in Sjogren's syndrome. Eur J Clin Invest.

[3] Ramos-Casals M, Brito-Zerón P, Sisó-Almirall A, Bosch X (2012). Primary Sjogren syndrome. BMJ.

[4] Gupta S, Gupta N (2017). Sjögren syndrome and pregnancy: a literature review. Perm J.

[5] Siamopoulou-Mavridou A, Manoussakis MN, Mavridis AK, Moutsopoulos HM (1988). Outcome of pregnancy in patients with autoimmune rheumatic disease before the disease onset. Ann Rheum Dis.

[6] Sarma A (2018). Hypokalemic paralysis due to primary Sjogren syndrome. Indian J Endocrinol Metab.

[7] Fox RI (2005). Sjögren's syndrome. Lancet.

[8] Michel L, Toulgoat F, Desal H, Laplaud DA, Magot A, Hamidou M, Wiertlewski S (2011). Atypical neurologic complications in patients with primary Sjögren's syndrome: report of 4 cases. Semin Arthritis Rheum.

[9] Lafitte C, Amoura Z, Cacoub P (2001). Neurological complications of primary Sjögren's syndrome. J Neurol.

[10] Urban E, Jabbari B, Robles H (1994). Concurrent cerebral venous sinus thrombosis and myeloradiculopathy in Sjögren's syndrome. Neurology.

[11] Ulivi L, Squitieri M, Cohen H, Cowley P, Werring DJ (2020). Cerebral venous thrombosis: a practical guide. Pract Neurol.

[12] de Seze J, Stojkovic T, Hachulla E (2001). Myelopathy - Sjogren's syndrome association: analysis of clinical and radiological findings and clinical course (Article in French). Rev Neurol (Paris).

[13] Tsokos M, Lazarou SA, Moutsopoulos HM (1987). Vasculitis in primary Sjögren's syndrome. Histologic classification and clinical presentation. Am J Clin Pathol.

[14] Massara A, Bonazza S, Castellino G (2010). Central nervous system involvement in Sjögren's syndrome: unusual, but not unremarkable--clinical, serological characteristics and outcomes in a large cohort of Italian patients. Rheumatology (Oxford).

[15] Fauchais AL, Lambert M, Launay D (2004). Antiphospholipid antibodies in primary Sjögren's syndrome: prevalence and clinical significance in a series of 74 patients. Lupus.

[16] Hughes GR, Khamashta MA (2003). Seronegative antiphospholipid syndrome. Ann Rheum Dis.

[17] Devreese KM, Zuily S, Meroni PL (2021). Role of antiphospholipid antibodies in the diagnosis of antiphospholipid syndrome. J Transl Autoimmun.

[18] Kiani IG, Qureshi SH, Shah F (2014). Depression and seizures as the main neuropsychiatric manifestation of mixed connective tissue disorder. J Coll Physicians Surg Pak.

